# *Plcg2^M28L^* Interacts With High Fat/High Sugar Diet to Accelerate Alzheimer’s Disease-Relevant Phenotypes in Mice

**DOI:** 10.3389/fnagi.2022.886575

**Published:** 2022-06-24

**Authors:** Adrian L. Oblak, Kevin P. Kotredes, Ravi S. Pandey, Alaina M. Reagan, Cynthia Ingraham, Bridget Perkins, Christopher Lloyd, Deborah Baker, Peter B. Lin, Disha M. Soni, Andy P. Tsai, Scott A. Persohn, Amanda A. Bedwell, Kierra Eldridge, Rachael Speedy, Jill A. Meyer, Johnathan S. Peters, Lucas L. Figueiredo, Michael Sasner, Paul R. Territo, Stacey J. Sukoff Rizzo, Gregory W. Carter, Bruce T. Lamb, Gareth R. Howell

**Affiliations:** ^1^Indiana University School of Medicine, Indianapolis, IN, United States; ^2^Department of Radiology & Imaging Sciences, Indiana University School of Medicine, Indianapolis, IN, United States; ^3^Stark Neurosciences Research Institute, Indianapolis, IN, United States; ^4^The Jackson Laboratory, Bar Harbor, ME, United States; ^5^Jackson Laboratory for Genomic Medicine, Farmington, CT, United States; ^6^Department of Medicine, Division of Clinical Pharmacology, Indiana University School of Medicine, Indianapolis, IN, United States; ^7^Department of Medicine, Aging Institute, University of Pittsburgh School of Medicine, Pittsburgh, PA, United States

**Keywords:** Alzheime’s disease, transcriptomics, diet, obesity, genetic risk alleles, predisposition

## Abstract

Obesity is recognized as a significant risk factor for Alzheimer’s disease (AD). Studies have supported the notion that obesity accelerates AD-related pathophysiology in mouse models of AD. The majority of studies, to date, have focused on the use of early-onset AD models. Here, we evaluate the impact of genetic risk factors on late-onset AD (LOAD) in mice fed with a high fat/high sugar diet (HFD). We focused on three mouse models created through the IU/JAX/PITT MODEL-AD Center. These included a combined risk model with *APOE4* and a variant in triggering receptor expressed on myeloid cells 2 (*Trem2^R47H^*). We have termed this model, LOAD1. Additional variants including the M28L variant in phospholipase C Gamma 2 (*Plcg2^M28L^*) and the 677C > T variant in methylenetetrahydrofolate reductase (*Mthfr*^677C >^ *^T^*) were engineered by CRISPR onto LOAD1 to generate LOAD1.*Plcg2^M28L^* and LOAD1.*Mthfr*^677C >^ *^T^*. At 2 months of age, animals were placed on an HFD that induces obesity or a control diet (CD), until 12 months of age. Throughout the study, blood was collected to assess the levels of cholesterol and glucose. Positron emission tomography/computed tomography (PET/CT) was completed prior to sacrifice to image for glucose utilization and brain perfusion. After the completion of the study, blood and brains were collected for analysis. As expected, animals fed a HFD, showed a significant increase in body weight compared to those fed a CD. Glucose increased as a function of HFD in females only with cholesterol increasing in both sexes. Interestingly, LOAD1.*Plcg2^M28L^* demonstrated an increase in microglia density and alterations in regional brain glucose and perfusion on HFD. These changes were not observed in LOAD1 or LOAD1.*Mthfr*^677C >^ *^T^* animals fed with HFD. Furthermore, LOAD1.*Plcg2^M28L^* but not LOAD1.*Mthfr*^677C >^ *^T^* or LOAD1 animals showed transcriptomics correlations with human AD modules. Our results show that HFD affects the brain in a genotype-specific manner. Further insight into this process may have significant implications for the development of lifestyle interventions for the treatment of AD.

## Introduction

Genetic and genome-wide association studies have identified variations in numerous genes that increase the risk for late-onset AD (LOAD). The E4 allele of apolipoprotein E (*APOE4*) is the greatest genetic risk factor but with variations in many other genes, including triggering receptor expressed on myeloid cells 2 (*TREM2*), also contribute to risk. Importantly, unlike relatively rare cases of familial AD (fAD), which are predominantly caused by mutations in *APP* or one APP processing gene, no single LOAD-associated variant is sufficient to cause AD. It is anticipated that combinations of genetic risk factors are required to develop LOAD. Studies also suggest that genetic factors may only contribute between 50 and 70% of the risk for LOAD, meaning many individuals are likely to develop LOAD due to a combination of genetic and environmental factors (Kawahara et al., 2017; Sarkar et al., 2019; Rahman et al., 2020; Kim et al., 2021). Studies have shown that diet, obesity, cardiovascular diseases, hypertension, physical activity, diabetes, educational attainment, smoking, and traumatic brain injury increase the risk for LOAD and other dementias (Ebbert et al., 2014; Baumgart et al., 2015; Xu et al., 2015; Wajman et al., 2018). Some of these risk factors can be attributed to a balance between diet and exercise. For instance, a western-style diet (e.g., a high fat/high sugar diet (HFD), and low in vitamins) in combination with a sedentary lifestyle can contribute to a metabolic syndrome, a cluster of conditions that includes increased blood pressure, high blood sugar, obesity, particularly excess body fat around the waist, and abnormal cholesterol or triglyceride levels. Metabolic syndrome increases the risk for Type 2 diabetes and cardiovascular disease, and AD (Kivipelto et al., 2001; Luchsinger et al., 2004; Cordain et al., 2005; Whitmer et al., 2008).

The Model Organism Development and Evaluation for Late-onset AD (MODEL-AD) consortium was established to develop mouse models that more faithfully develop LOAD-relevant phenotypes compared to previous mouse models that were largely based on fAD. Two centers, one from the Indiana University (IU), the Jackson Laboratory (JAX), the University of Pittsburgh (PITT), and Sage Bionetworks, and the other from the University of California Irvine (UCI), have focused on incorporating genetic risk factors into C57BL/6J (B6) mice and assaying human-relevant phenotypes. Initially, the IU/JAX/PITT group created mice that are double homozygous for *APOE4* and *Trem2^R47H^* (termed LOAD1) (Kotredes et al., 2021). The *APOE4* is the greatest genetic risk factor for LOAD which is thought to increase the risk for AD through both gain and loss of function effects on multiple processes in the brain including amyloid clearance, synaptic plasticity, and cerebrovascular health (Liu et al., 2013). The *TREM2^R47H^* is a rare coding mutation with a partial loss of function that alters the behavior of microglia (Carmona et al., 2018). LOAD1 mice and controls were aged 24 months and data showed age but not genotype was the major factor-driving changes in LOAD1 mice (Kotredes et al., 2021). Therefore, these mice provided a sensitized background for assessing additional genetic and environmental risk factors (Kotredes et al., 2021). To further sensitize LOAD1 mice, additional LOAD genetic risk factors were added *via* CRISPR/CAS9. These included the M28L variant in phospholipase C Gamma 2 (*Plcg2^M28L^*) (Tsai et al., 2021) and the 677C > T variant in methylenetetrahydrofolate reductase (*Mthfr*^677C >^ *^T^*) (Wang et al., 2005; Chan et al., 2019). The PLCG2 gene encodes an enzyme that catalyzes the conversion of phospholipid PIP2 (1-phosphatidyl-1D-myo-inositol 4,5-bisphosphate) to IP3 (1D-myo-inositol 1,4,5-trisphosphate) and DAG (diacylglycerol). PLCG2 plays a crucial role in signal transduction between tyrosine kinases and downstream events, protein kinase C activation, and intracellular calcium release (Hernandez et al., 1994). In the CNS, PLCG2 is involved in TREM2/TYROBP signaling pathway and is selectively expressed in microglia, playing important roles in inflammation, phagocytosis, and lipid sensing. Although the precise effects of the M28L variant have not yet been elucidated, like TREM2, PLCG2 is expressed by microglia and plays a role in amyloid clearance (Carmona et al., 2018). MTHFR functions in the folate/methionine/homocysteine pathway are expressed both peripherally and centrally, and the *MTHFR*^677C >^ *^T^* variant causes an increase in homocysteine increasing the risk for a variety of diseases including cardiovascular and cerebrovascular-related disorders and AD (Liu et al., 2010; Liew and Gupta, 2015; Rai, 2017). Transcriptomic analyses of the brain tissue from LOAD1.*Plcg2^M28L^* or LOAD1.*Mthfr*^677C >^ *^T^* mice revealed increased alignment to brain transcriptomes from human patients with AD compared to B6 or LOAD1 controls (Preuss et al., 2020).

Despite carrying multiple genetic risk variants for LOAD, LOAD1, LOAD1.*Plcg2^M28L^*, and LOAD1.*Mthfr*^677C >^ *^T^* did not natively recapitulate all phenotypes relevant to LOAD, making these strains ideal to test the effects of environmental risk factors, such as diet. Commonly, the diet consumed by the western world is high in fat and refined sugar. This has led to a global obesity epidemic where, for instance, 42% of all Americans are considered overweight (The Center For Disease Control And Prevention, 2022). Obesity can cause a wide range of changes including increased inflammation in both the periphery and the brain (Ellulu et al., 2017; Chan et al., 2019). Inflammation is characterized, in part by increased production of cytokines, such as IL1b and the activation of myeloid cells, including microglia in the brain (Wang et al., 2015). Chronic inflammation has been shown to greatly increase the risk for LOAD (Furman et al., 2019).

Multiple studies in mice have assessed the effects of a western-like diet in the context of aging and AD. For instance, a study showed a diet-induced myelin breakdown in aging B6 mice that were dependent on the complement cascade (Graham et al., 2020). Also, the addition of an HFD exacerbated AD phenotypes in mouse models relevant to early-onset AD (Knight et al., 2014; Jones et al., 2019; Bracko et al., 2020; Robison et al., 2020; Jones et al., 2021). In one study, Jones et al. (2019) found that with HFD, male *APOE4* mice were more susceptible to metabolic disturbances, including glucose intolerance when compared to *APOE3* mice. Behavioral deficits were not observed due to HFD, suggesting that metabolic responses to HFD are dependent on both sex and *APOE* genotype. A second study concluded that early dysregulation of inflammation in *APOE4* brains could predispose to CNS damage from various insults, including diet, and later results in the increased CNS damage normally associated with the *APOE4* genotype (Jones et al., 2021). However, the effects of a western-like diet in the context of multiple genetic risk factors for LOAD have not been studied. To address this, a commonly used HFD (high in fat and sugar, refer to the section, Methods) was fed to male and female LOAD1, LOAD1.*Plcg2^M28L^*, and LOAD1.*Mthfr*^677C >^ *^T^* mice from 2 to 12 months. Biometric measures were collected throughout the study, *in vivo* imaging to assess glucose utilization and blood perfusion in a region-specific manner in the brain was carried out prior to perfusion, and brain transcriptomics, neuropathological assessments, and protein quantification were performed postmortem. Results showed that the effects of consumption of HFD to midlife were not uniform across all models but dependent on specific genetic risk factors. In particular, the effects of the HFD were most severe in the presence of *Plcg2^M28L^*.

## Materials and Methods

### Animal Housing Conditions at Indiana University and the Jackson Laboratory

All animals were obtained from the JAX and are congenic to the C57BL/6J (JAX# 000664) (B6) strain. LOAD1 is homozygous for both *APOE4* and *Trem2^R47H^* (JAX ID:28709). B6.*Plcg2^M28L^*/*APOE4*/*Trem2^R47H^* (triple homozygous, LOAD1.*Plcg2^M28L^*, JAX ID:30674) was created using CRISPR/Cas9 to introduce the M28L LOAD risk variant into LOAD1 mice.B6.*Mthfr*^677C >^ *^T^*/*APOE4*/*Trem2^R47H^* (triple homozygous, LOAD1.*Mthfr*^677C >^ *^T^*, JAX ID:30922), was also created using CRISPR/Cas9 to introduce the 677TC > T variant into LOAD1 mice (Reagan et al., 2021). All strains were validated using brain RNA-seq to confirm no off-target effects and no alterations in target transcript isoforms/expression levels (Kotredes et al., 2021). More details on strain creation are provided on the AD Knowledge Portal.^1^

The effects of HFD on LOAD1.*Plcg2^M28L^* mice were assessed at the Indiana University (IU), with LOAD1.*Mthfr*^677C >^ *^T^* mice assessed at JAX. LOAD1 mice acted as site-matched controls. An additional cohort of B6 mice was assessed at IU as a strain control. At IU, for experimental cohorts, LOAD1.*Plcg2^M28L/^*^+^ mice were intercrossed to create LOAD1.*Plcg2^M28L^* triple homozygous and LOAD1 litter-matched control mice. At JAX, for experimental cohorts, LOAD1.*Mthfr*^677TC > *T/*+^ mice were intercrossed to create LOAD1.*Mthfr*^677TC >^ *^T^* triple homozygous and LOAD1 litter-matched control mice. Up to five mice were housed per cage with SaniChip bedding and were initially provided with LabDiet^®^ 5K52/5K67 (6% fat, control diet, CD^2^). Mouse rooms were kept on a 12:12 light:dark schedule with the lights on from 7:00 a.m. (6:00 a.m. at JAX) to 7:00 p.m. daily (6:00 p.m. at JAX). Mice were initially ear-punched for identification and then following genotype confirmations, microchipped using a p-chip system (PharmaSeq), with the microchips placed at the base of the tail. At 2 months of age (mos), experimental cohorts were randomized into two groups: Group 1 continued on CD *ad libitum* and Group 2 was provided with ResearchDiet^®^ feed D12451i (45% of high fat; 35% of carbohydrates, HFD^3^) *ad libitum*. All procedures were approved by the IU or JAX Institutional Animal Care and Use Committees (IACUC). Where possible, all housing and procedures were standardized and aligned across sites. Unless specified, n = 10 for each sex, genotype, and diet were used.

Mice were anesthetized to the surgical plane of anesthesia with tribromoethanol at 12 months of age. Under complete anesthesia, animals were euthanized by decapitation and perfused through the heart with ice-cold phosphate-buffered saline (PBS). Blood and trunk blood was centrifuged for 15–20 min at 4°C at 14,500 RPM, and the plasma was stored at –80°C.

### Blood Plasma Analysis, Perfusion, and Preparation of Tissue Samples

Blood was collected longitudinally from fasting mice at 8 months of age *via* a cheek puncture and again at the terminal timepoint of 12 months of age where mice were anesthetized, and blood was extracted through a left ventricle cardiac puncture with a 25 g of EDTA-coated needle before PBS perfusion. Brain tissue was collected immediately after euthanasia. Approximately 500 mL of whole blood was transferred to a MAP-K2 EDTA Microcontainer (BD, Franklin Lakes, NJ, United States) on ice and centrifuged at 4°C at 4388 × *g* in a pre-chilled ultracentrifuge for 15 min. Without disturbing the red blood cell fraction, serum supernatant was pipetted into a chilled cryovial with a p200 tip and immediately snap-frozen on dry ice for 10 min. Samples were stored at –80°C and later thawed for the analysis of glucose, total cholesterol, low-density lipoprotein (LDL), high-density lipoprotein (HDL), triglyceride, and non-essential fatty acid (NEFA) levels with the Siemens Advia 120 (Germany).

### Histology and Fluorescent Immunostaining

After perfusion and dissection, the left side of each mouse brain was fixed in 4% of paraformaldehyde. After a transfer to 10% sucrose the following day, the brains were transferred to 30% of sucrose for storage. Brains were sectioned, coronally (Jax) and sagittally (IU), at 10–20 μm on a freezing microtome.

#### NeuN Staining

Sections were washed and blocked for 1 h in 10% of host goat serum. Sections were incubated overnight at 4°C in a solution containing the antibody, NeuN rabbit (ab104225, 1:1,000, Abcam). After additional washes, the sections were incubated for 1 h at room temperature in a secondary solution containing the fluorescent markers, goat anti-rabbit 488 (A11034, 1:1,000, Invitrogen). After one additional wash, the sections were mounted on charged slides, counterstained, and coverslipped with Prolong Gold Antifade Mountant with DAPI.

#### Iba1 Staining

Sections were washed and then blocked with 10% of host goat serum for 2 h. Sections were incubated overnight at 4°C in a solution containing the Recombinant Anti-Iba1 antibody rabbit (ab178847, 1:500, Abcam). After additional washes, the sections were incubated for 1 h at room temperature in a secondary solution containing the fluorescent markers, goat anti-rabbit 488 (A11034, 1:1000, Invitrogen). After one additional wash, the sections were mounted on charged slides, counterstained, and coverslipped with Prolong Gold Antifade Mountant with DAPI.

Microscopy was used to view and capture images of the immunofluorescent stains with a Leica DM6 B and DFC7000 GT camera using Leica Microsystems’ LAS X software. Further image capture and image scanning were performed on an Andor Zyla 5.5 sCMOS camera with an Aperio Versa scanner and Versa software. One to three coronal (LOAD1.*Mthfr*^677C >^ *^T^*) or sagittal (LOAD1.*Plcg2^M28L^*) sections were analyzed with Imaris software to generate density measurements within the cortex and subiculum (Supplementary Figure 1A).

### RNA Extraction and Nanostring Analysis

As previously described (Preuss et al., 2020; Oblak et al., 2021), total RNA was extracted from frozen right brain hemispheres using the MagMAX mirVana Total RNA Isolation Kit (Thermo Fisher Scientific) and the KingFisher Flex purification system (Thermo Fisher Scientific, Waltham, MA; n = 6 per sex/genotype/diet). RNA concentration and quality were assessed using the Nanodrop 2000 spectrophotometer (Thermo Fisher Scientific) and the RNA Total RNA Nano assay (Agilent Technologies, Santa Clara, CA, United States). The NanoString Mouse AD gene expression panel was used for gene expression profiling on the nCounter platform (NanoString, Seattle, WA, United States) as described by the manufacturer. The nSolver software was used for generating raw NanoString gene expression values. The NanoString data were normalized by dividing raw counts within a lane by the geometric mean of the housekeeping genes from the same lane (Preuss et al., 2020). Next, normalized count values were log-transformed for downstream analysis.

The NanoString gene expression data were generated for male and female LOAD1, LOAD1.*Plcg2^M28L^*, and LOAD1.*Mthfr*^677C >^ *^T^* mice fed on standard chow diet and HFD. We assessed the effects of sex, HFD, each genetic variant (*Mthfr*^677C >^ *^T^*, *Plcg2^M28L^*), the interaction between HFD and *Mthfr*^677C >^ *^T^*, and the interaction between HFD and *Plcg2^M28L^*. To determine the effects of each of these factors, we fit a multiple regression model using the lm function in R as (Pandey et al., 2019):


$$L\,o\,g\,\left(e\,x\,p\,r\right)=\beta_{0}+\sum_{i}\beta_{i}+\varepsilon$$

Where the sum is over sex (male), HFD, genetic variants (*Mthfr*^677C >^ *^T^*, *Plcg2^M28L^*), and interaction terms between HFD and each variant (HFD*Mthfr^677C >^ *^T^*, HFD*Plcg2^M28L^). The log(expr) represents the log-transformed normalized count from the NanoString gene expression panel (Preuss et al., 2020). In this formulation, the standard chow diet and LOAD1 genetic background serve as controls. The values, β_0_ and ε represent the average expression level for the reference LOAD1 male mice on the standard chow diet and residual, respectively.

Next, we have computed Pearson’s correlation between gene expression changes (log-fold change) in human cases with AD vs. controls in each AMP-AD module (Greenwood et al., 2020) and the effect of each mouse perturbation (sex, HFD, *Mthfr*^677C >^ *^T^*, *Plcg2^M28L^*, and interaction terms HFD*Mthfr^677C >^ *^T^*, HFD*Plcg2^M28L^) as measured above for each gene in NanoString panel (Pandey et al., 2019; Preuss et al., 2020) using cor.test function built in R as:


c⁢o⁢r.t⁢e⁢s⁢t⁢(L⁢o⁢g⁢F2⁢C⁢(A⁢D/c⁢o⁢n⁢t⁢r⁢o⁢l),β)

from which we obtained both the correlation coefficient and the significance level (*p*-value) of the correlation. Log_2_FC values for human transcripts were obtained through the AD Knowledge Portal (Greenwood et al., 2020).^4^

Similarly, we have computed Pearson’s correlation between gene expression changes (log-fold change) in human AD cases versus controls in each of the LOAD subtypes (Milind et al., 2020) and the effect of each mouse perturbation (sex, HFD, *Mthfr*^677C >^ *^T^*, *Plcg2^M28L^*, interaction terms HFD*Mthfr^677C >^ *^T^*, and HFD*Plcg2^M28L^) for each gene in Nanostring panel (Pandey et al., 2019; Preuss et al., 2020) using cor.test function built in R as:


c⁢o⁢r.t⁢e⁢s⁢t⁢(L⁢o⁢g⁢F2⁢C⁢(L⁢O⁢A⁢D⁢S⁢u⁢b⁢t⁢y⁢p⁢e/c⁢o⁢n⁢t⁢r⁢o⁢l),β)

from which we obtained both the correlation coefficient and the significance level (*p*-value) of the correlation. Here, Log_2_FC(LOAD Subtype/control) represents the log-fold change in gene expression in each subtype vs. control.

We plotted the correlation results using the ggplot2 package in R. Circles within a square correspond to significant (*p* < 0.05) positive (blue) and negative (red) Pearson’s correlation coefficients. The color intensity and size of the circles are proportional to Pearson’s correlation coefficient.

### Human Post-mortem Brain Cohorts and Gene Co-expression Modules

Wan et al. (2020) discovered 30 human brain co-expression modules based on the meta-analysis of differential gene expression from seven distinct regions: the dorsolateral prefrontal cortex (DLPFC), superior temporal gyrus (STG), frontal pole (FP), parahippocampal gyrus (PHG), temporal cortex (TCX), inferior frontal gyrus (IFG), and the cerebellum (CBE) in postmortem samples obtained from three independent LOAD cohorts: the Religious Orders Study and the Memory and Aging Project (ROSMAP) cohort, the Mount Sinai Brain Bank (MSBB), and the Mayo Clinic cohort (Allen et al., 2016; De Jager et al., 2018; Wang et al., 2018). Briefly, Wan et al. (2020) performed library normalization and covariate adjustments for each human study separately using fixed/mixed effects modeling to account for batch effects. Initially, a total of 2,978 brain regions’ specific expression modules were identified across all tissues (doi: 10.7303/syn10309369.1) using five distinct network module identification algorithms (MEGENA, WINA, metanetwork, rWGCNA, and speakEasy). Next, 660 modules were selected that were significantly enriched for at least one AD-specific differentially expressed gene set from the meta-analysis. Lastly, the graph clustering method was applied to identify 30 aggregate modules that are not only differentially expressed but are also replicated across multiple independent co-expression module algorithms (Wan et al., 2020). Wan et al. classified these 30 aggregate co-expression modules into five distinct consensus clusters (Wan et al., 2020). These consensus clusters consist of a subset of modules that are associated with similar AD-related changes across the multiple studies and brain regions. Reactome pathway^5^ enrichment analysis was used to identify specific biological themes across these five consensus clusters. Pathways were ranked based on their Bonferroni corrected *p*-values to account for multiple testing. Finally, consensus clusters were annotated based on the highest ranked and non-overlapping term for each functionally distinct cluster.

### Human LOAD Subtypes

Milind et al. (2020), integrated post-mortem brain gene co-expression data from the frontal cortex, temporal cortex, and hippocampus due to their relevance to LOAD neuropathology across independent human cohorts (ROS/MAP, Mount Sinai Brain Bank, and Mayo Clinic) and stratified patients into different molecular subtypes based on gene co-expression profiles using iterative WGCNA (Allen et al., 2016; De Jager et al., 2018; Wang et al., 2018). Two distinct LOAD subtypes were identified in the ROSMAP cohort, three LOAD subtypes were identified in the Mayo cohort, and two distinct LOAD subtypes were identified in the MSBB cohort. Similar subtype results were observed in each cohort, with LOAD subtypes found to primarily differ in their inflammatory response based on differential expression analysis (Milind et al., 2020).

### Kyoto Encyclopedia of Genes and Genomes Pathway Enrichment Analysis

Kyoto Encyclopedia of Genes and Genomes (KEGG) pathway enrichment analysis was performed using clusterprofiler package (Yu et al., 2012) within the R software environment. Pathways were determined to be significant after multiple testing corrections (FDR adjusted *p* < 0.05). Gene set enrichment analysis (GSEA) was used based on the method proposed by Subramanian et al. (2005) as implanted in the clusterProfiler package for the KEGG pathway library. Nanostring AD panel genes were ranked based on regression coefficients calculated for each factor (sex, high fat diet, *Mthfr*^677C >^ *^T^*, *Plcg2^M28L^*, interaction terms HFD*Mthfr^677C >^ *^T^*, and HFD*Plcg2^M28L^). Enrichment scores for all KEGG pathways were computed to compare relative expression on the pathway level between each factor. Principal component analysis (PCA) was performed using the resulting gene set normalized enrichment scores (NES).

### *In vivo* PET/CT Imaging

To assess regional glycolysis and tissue perfusion, mice were non-invasively imaged *via* PET/CT (n = 10 mice/sex/genotype/age). To measure the regional blood flow, copper-pyruvaldehyde-bis(N4-methylthiosemicarbazone) (^64^ Cu-PTSM)(Green, 1987), which has a very high first pass (>75%) extraction (Mathias et al., 1991) and glutathione reductase redox trapping of copper (Mathias et al., 1991), was administered *via* tail vein in awake subjects and was given a 2 min uptake period prior to imaging. To measure regional glycolytic metabolism, 2-fluoro-2-deoxyglucose (^18^F-FDG) was administered *via* tail vein in awake subjects and given a 30 min uptake period prior to imaging. Post uptake, mice were induced with 5% of isoflurane (95% medical oxygen) and maintained during acquisition with 1–2% of isoflurane at 37°C. To provide both anatomical structure and function, PET/CT imaging was performed with a Molecubes b-X-CUBE system (Molecubes NV, Gent Belgium). For PET determination of blood flow and metabolism, calibrated listmode PET images were acquired on the b-CUBE and reconstructed into a single-static image using ordered subset expectation maximization (OSEM) with 30 iterations and 3 subsets (Krishnamoorthy et al., 2018). To provide anatomical reference and attenuation maps necessary to obtain fully corrected quantitative PET images, helical CT images were acquired with tube voltage of 50 kV, 100 mA, 100 μm slice thickness, 75 ms exposure, and 100 μm resolution. In all cases, images were corrected for radionuclide decay, tissue attenuation, detector dead-time loss, and photon scatter according to the manufacturer’s methods (Krishnamoorthy et al., 2018). Post-acquisition, all PET and CT images were co-registered using a mutual information-based normalized entropy algorithm (Studholme et al., 1998) with 12 degrees of freedom and mapped to stereotactic mouse brain coordinates (Franklin and Paxinos, 2013). Finally, to quantify regional changes, voxels of interest (VOIs) for 27 brain (54 bilateral) regions were extracted and analyzed for standardized uptake value ratios (SUVRs) according to published methods (Dandekar et al., 2007).

#### Autoradiography

To provide secondary confirmation of the *in vivo* PET images, and to quantify tracer uptake regionally, the brains were extracted post rapid decapitation, gross sectioned along the midline, slowly frozen on dry ice, then embedded in cryomolds with optimal cutting temperature (OCT) compound (Tissue-Tek). Thin frozen sections (20 μm) were obtained *via* cryotomy at prescribed bregma targets (n = 6 bregma/mouse, 6 replicates/bregma) according to stereotactic mouse brain coordinates (Franklin and Paxinos, 2013). Sections were mounted on glass slides, air dried, and exposed on BAS Storage Phosphor Screens (SR 2040 E, Cytiva Inc.) for up to 12 h. The post-exposure, screens were imaged *via* Typhoon FL 7000IP (GE Medical Systems) phosphor-imager at 25 μm resolution along with custom 18F or ^64^Cu standards described previously (Territo et al., 2017).

#### Image Analysis

All PET and MRI images were co-registered using a ridged-body mutual information-based normalized entropy algorithm (Studholme et al., 1997) with 12 degrees of freedom and mapped to stereotactic mouse brain coordinates (Franklin and Paxinos, 2013) using MIM 7.0.5 (MIM Software Inc., Beachwood, OH, United States). Post-registration, 56 bilateral regions were extracted *via* brain atlas and averaged to yield 27 unique volumes of interest that map to key cognitive and motor centers that include the agranular insular cortex, auditory cortex, caudate putamen, cerebellum, cingulate cortex, corpus callosum, dorsolateral orbital cortex, dorsintermed entorhinal cortex, dysgranular insular cortex, ectorhinal cortex, fornix, frontal association cortex, hippocampus, lateral orbital cortex, medial orbital cortex, parietal cortex, parietal association cortex, perirhinal cortex, prelimbic cortex, primary motor cortex, primary somatosensory cortex, retrosplenial dysgranular cortex, secondary motor cortex, secondary somatosensory cortex, temporal association cortex, thalamus, ventral orbital cortex, and visual cortex. For autoradiographic analysis, tracer uptake was quantified on hemi-coronal sections by manually drawing regions of interest for 17 regions of interest (i.e., the auditory cortex, caudate putamen, cerebellum, cingulate cortex, corpus callosum, dorso-intermed entorhinal cortex, dysgranular insular cortex, ectorhinal cortex, hippocampus, hypothalamus, medial septum, primary motor cortex, primary somatosensory cortex, retrosplenial dysgranular cortex, temporal association cortex, thalamus, and visual cortex) on calibrated phosphor screen at bregma 0.38, -1.94, and -3.8 mm using MCID (InterFocus Ltd.). To permit dose and brain uptake normalization, SUVRs relative to the cerebellum were computed for PET and autoradiograms for each subject, genotype, and age as follows:


$$S\,U\,V\,R\,\left(s,R,g,a\right)=\frac{R\,\left(s,g,a\right)}{C\,\left(s,g,a\right)}$$

where, *s*, *g*,*a*, *R*, and *C* are the subject, genotype, age, region/volume of interest, and cerebellum region/volume of interest. In all cases, the region/volumes of interest were subjected to PCA analysis, and regions that explain the top 80% of the variance were analyzed for differences with time and genotype using a two-way ANOVA (Prism, GraphPad Inc.), where significance was taken at *p* < 0.05.

### Homogenization and Protein Extraction

Each hemibrain was weighed prior to homogenizing in tissue protein extraction reagent (T-PER Thermo Fisher Scientific; 1 mL per 100 mg of tissue weight) supplemented with protease and phosphatase inhibitors cocktail (Sigma-Aldrich). Total protein concentration was measured using bicinchoninic acid (BCA; Pierce). Hemibrain lysates were then aliquoted and kept in the –80°C freezer for long-term storage. The primary supernatant was utilized to analyze the content of proinflammatory cytokines.

### Cytokine Panel Assay

Mouse hemibrain samples were assayed in duplicate using the MSD Proinflammatory Panel I (K15048D; MesoScale Discovery, Gaithersburg, MD, United States), a highly sensitive multiplex enzyme-linked immunosorbent assay (ELISA). This panel quantifies the following 10 proinflammatory cytokines in a single small sample volume (25 μL) of supernatant using an electrochemiluminescent detection method (MSD): interferon γ (IFN-γ), interleukin (IL)-1β, IL-2, IL-4, IL-6, IL-8, IL-10, IL-12p70, IL-13, and tumor necrosis factor α (TNFα). The mean intra-assay coefficient for each cytokine was less than 8.5%, based on the cytokine standards. Any value below the lower limit of detection (LLOD) for the cytokine assay was replaced with ½ LLOD of the assay for statistical analysis.

### Data Analysis and Study Design

For biometric studies where multiple samples were collected at multiple time points for a mouse, a mixed ANOVA for repeated measures was done. For IHC and cytokine analyses, a two-way ANOVA was completed, followed by Tukey’s *post hoc* testing. For these studies, *p* < 0.05 was considered significant. Multiple cohorts of animals were utilized in this study for different outcome measures, a summary of mouse numbers can be found in Supplementary Figure 1B.

## Results

### Chronic Consumption of HFD-Induced Features of Metabolic Syndrome

**FIGURE 1:**
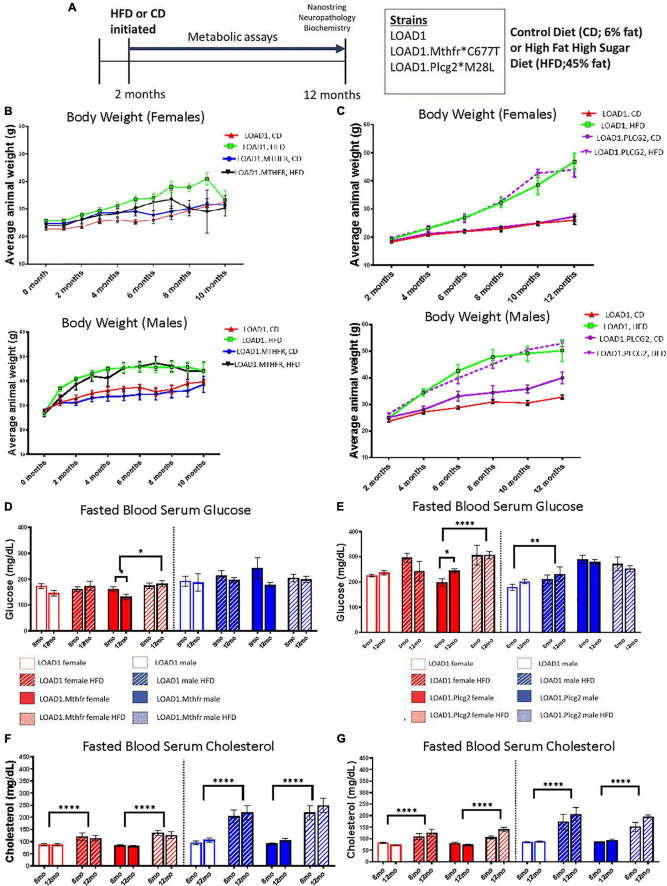
Bodyweight, glucose, and cholesterol increased on a high fat/high sugar (HFD) diet, regardless of genotype. LOAD1, LOAD1.*Mthfr*^677C >^ *^T^*, and LOAD1.*Plcg2^M28L^* mice were fed with a control diet (CD) or HFD from 2 to 12 months of age **(A)**. At multiple timepoints throughout the study, blood was collected. At the terminal time point, transcriptomics, limited neuropathology, and biochemical studies were completed. Over the course of the study, regardless of genotype or sex, mice fed with HFD gained more weight than animals fed with a control diet **(B)**. Glucose **(D,E)** and Cholesterol **(F,G)** increased over time, which was related to diet, but not to genotype **(C–F)**. A mixed ANOVA with repeated measures was completed; *p* < 0.05 is considered significant. *p < 0.05, **p < 0.01, ****p < 0.0001.

This study consisted of three mouse strains, LOAD1, LOAD1.*Mthfr^677C^*^>^ *^T^*, and LOAD1.*Plcg2^M28L^* on either a normal control diet (6%, CD) or an HFD (45%). LOAD1.*Plcg2^M28L^* and LOAD1 mice were assessed at IU and LOAD1.*Mthfr*^677C >^ *^T^* and LOAD1 mice were assessed at JAX. LOAD1 acted as site-matched controls. For both studies, HFD was introduced at 2 months of age in half of the animals, while the other half was maintained on CD (Figure 1A, refer to Methods for breeding strategies). Bodyweight was monitored once a month for the LOAD1.*Mthfr*^677C >^ *^T^* study (Figure 1B) and once every two months for the LOAD1.*Plcg2^M28L^* study (Figure 1C). Mice fed on HFD gained more weight compared to those fed on CD, irrespective of genotype (Figures 1B,C). These data are consistent with the induction of diet-induced obesity (DIO) that has been previously reported for B6 mice (Wanrooy et al., 2018). Blood samples were collected from fasting, non-anesthetized mice at 8 (mid stage) and 12 (end stage) months of age to determine glucose and total cholesterol levels (Figures 1D–G). In general, blood glucose levels were slightly lower in all mice housed at JAX (Figure 1D) compared to IU (Figure 1E) which may be due to differences in environment, despite efforts to standardize housing conditions. In both studies, there was a significant effect of HFD in female, but not male mice fed on HFD. The effect was more striking in female mice from IU (*p* < 0.0001) compared to female mice from JAX (*p* = 0.165). Fasting blood glucose levels only exceeded 240 mg/dL (a level that previous studies consider B6 mice to be diabetic (Xiang et al., 2021) in mice fed with LOAD1 HFD and LOAD1.*Plcg2^M28L^* HFD. Fasting serum cholesterol levels were consistently elevated at both 8 and 12 months of age in all strains fed with an HFD compared to those fed with CD independent of genotype and sex (*p* > 0.0001 at JAX, *p* > 0.0001 at IU; Figures 1F–G). Collectively, these data suggest HFD-induced features of some metabolic syndrome, potentially in a sex-specific manner, but independent of genotype.

### Neuron Number Was Unchanged but Reactive Microglia Increased in LOAD1.*Plcg2^M28L^* Animals

**FIGURE 2:**
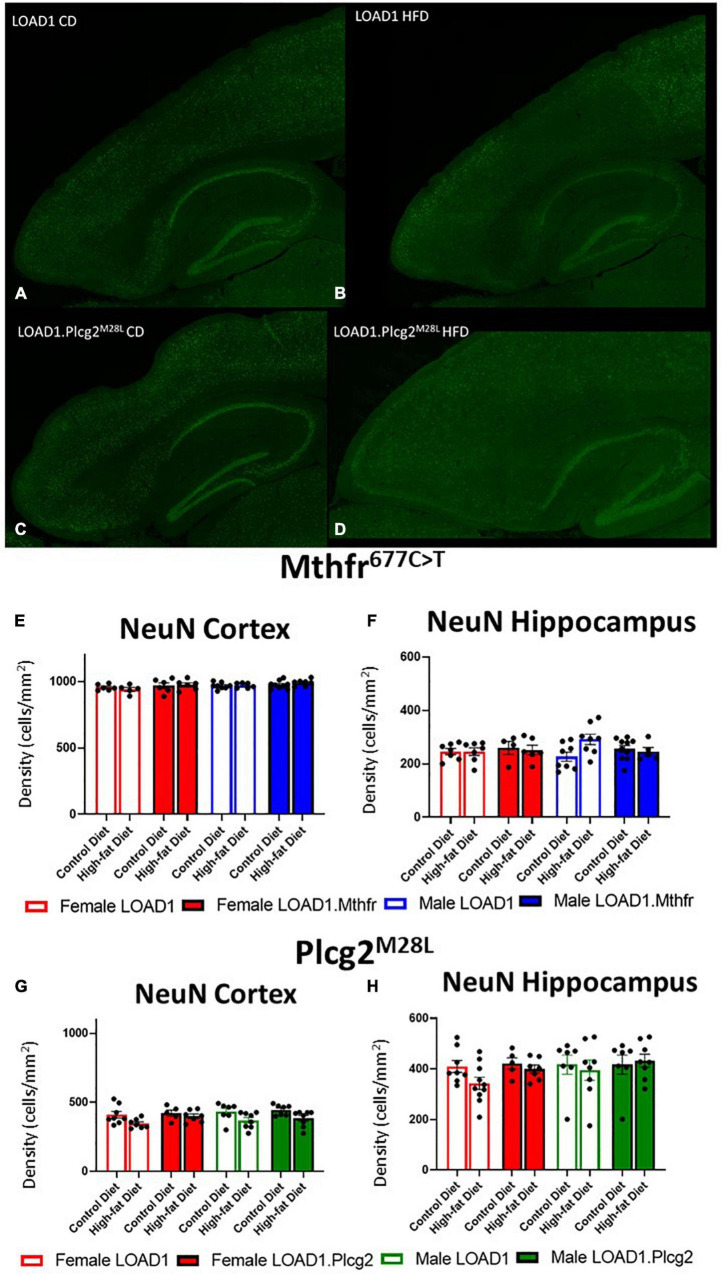
Neuron density is unaffected by HFD in LOAD1.*Plcg2^M28L^* and LOAD1.*Mthfr*^677C >^ *^T^* mice. Immunohistochemistry was completed using NeuN to visualize the density of neurons **(A–D)** in the LOAD1.*Plcg2^M28L^* mice. The HFD has no significant effect on the density of neurons in either the cortex **(E,G)** or the hippocampus **(F,H)** of either LOAD1.*Plcg2^M28L^* or LOAD1.*Mthfr*^677C >^ *^T^* mice. Statistical analysis was completed using an ANOVA followed by Tukey’s *post hoc* tests. *P* < 0.05 is considered significant.

**FIGURE 3:**
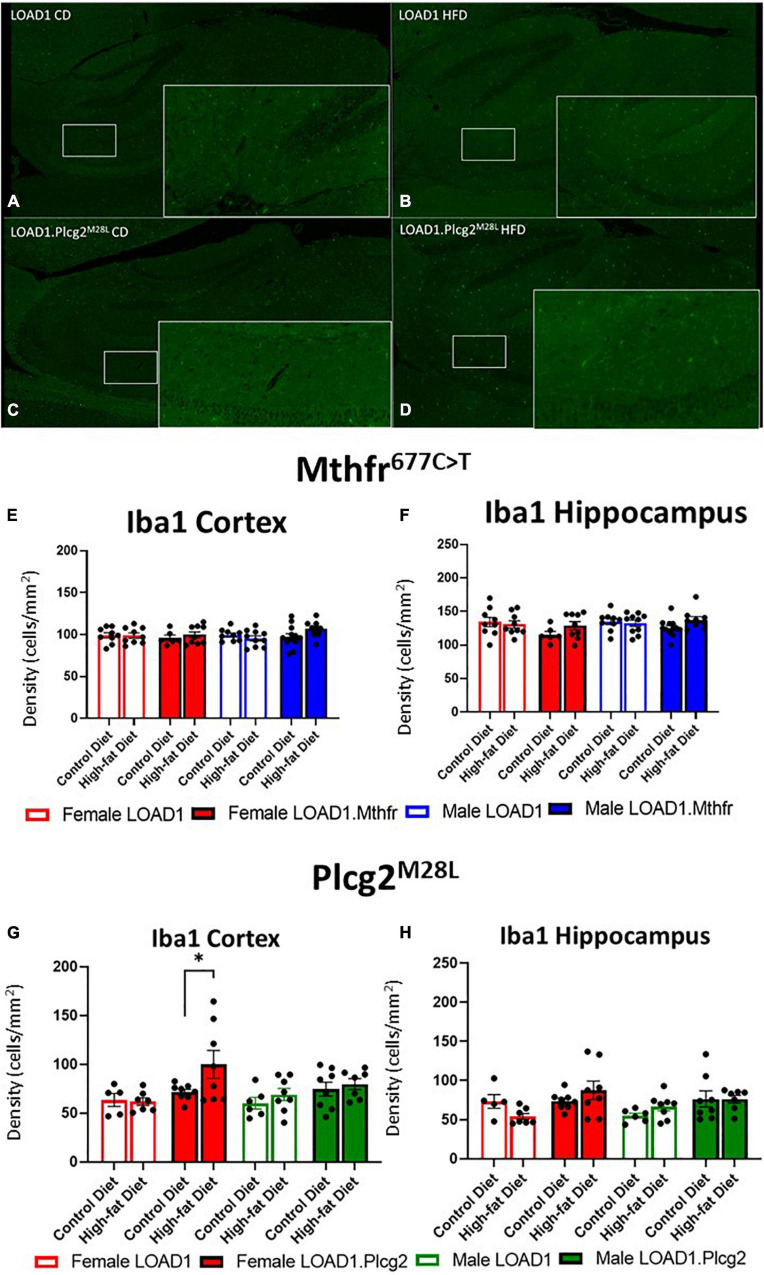
Microglial density is increased in LOAD1.*Plcg2^M28L^* high fat diet mice. Immunohistochemistry was completed using Iba1 to visualize the density of microglia **(A–D)** in the LOAD1.*Plcg2^M28L^* mice. LOAD1.*Mthfr*^677C >^ *^T^* mice did not show any significant changes in microglia density in the cortex **(E)** or hippocampus **(F)**, regardless of diet. However, female LOAD1.*Plcg2^M28L was^* found to have an increase in the microglial density in the cortex of the mice fed with HFD **(G)**. No changes were observed in LOAD1.*Plcg2^M28L^* mice fed with the control diet (CD) **(G,H)**. Data suggest a gene by diet interaction that may be sex-specific. Statistical analysis was completed using an ANOVA followed by Tukey’s *post hoc* tests. *P* < 0.05 is considered significant.

A hallmark of LOAD and other dementias is the loss of neurons, particularly in the cortex and hippocampus. However, the cortical and hippocampal neuronal cell loss is largely absent in the most widely used mouse models of fAD and LOAD. Neuronal cell loss was also not observed in LOAD1 mice, even at 24 months of age (Kotredes et al., 2021). To assess the neuronal cell density in this study, we examined both the cortex and the hippocampus of CD and HFD animals from both LOAD1.*Mthfr*^677C >^ *^T^* and LOAD1.*Plcg2^M28L^* animals and controls utilizing fluorescent immunostaining (Figures 2A–D and Supplementary Figure 1). In both LOAD1.*Mthfr^C677T^* and LOAD1.*Plcg2^M28L^* animals, there was no quantitative difference in the density of neurons in either genotype when comparing the CD to the HFD (Figure 2). The second hallmark of LOAD is neuroinflammation, particularly microglia activation. In our previous study, LOAD1 mice did not show microglia activation. When analyzing the number of IBA1+ cells in the brain, compared to LOAD1 mice either on the CD or HFD, we found increased IBA1+ cells in the cortex of female LOAD1.*Plcg2^M28L^* mice fed on HFD compared to LOAD1.*Plcg2^M28L^* on CD (Figure 3 and Supplementary Figure 1). These data are not collected based on stereological analyses and are therefore suggestive of a reduced density of IBA1+ cells. Interestingly, we did not see a difference in LOAD1.*Mthfr*^677C >^ *^T^* mice (Figure 3), suggesting an HFD-induced neuroinflammatory reaction that is specific to the LOAD1.*Plcg2^M28L^* genotype.

### Plasma Cytokines and Brain Cytokines Are Altered in HFD Animals

**FIGURE 4:**
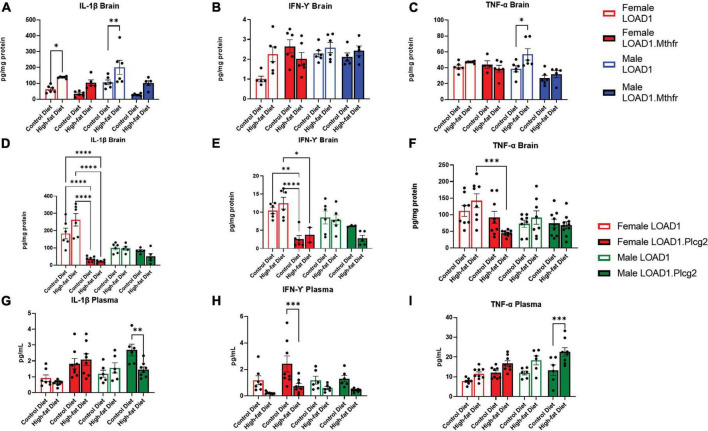
Cytokine production is altered in mice fed with HFD. In order to identify peripheral factors that may be driving the genetic by diet interaction, we examined the brain **(A–F)** and plasma **(G–I)** cytokines in both LOAD1.*Plcg2^M28L^* or LOAD1.*Mthfr^677C >^ *^T^** mice. In LOAD1 mice, significant increases in brain IL-1β **(A)** and TNF-a **(C)** were observed in mice fed with HFD; however, LOAD1.*Mthfr^677C >^ *^T^** mice did not have any significant increases. In the LOAD1.*Plcg2^M28L^* mice, a significant decrease in brain IL-1b **(D)** and IFN-g **(E)** was observed in the females, regardless of diet. The HFD did not show the same increase in the brain cytokine levels as the control (LOAD1) animals, suggesting a deficiency in cytokines due to the variant. TNF-a was reduced in females as well **(F)**, but only in the HFD group. In the plasma of LOAD1.*Plcg2^M28L^* mice, we observed a reduction in IL-1b **(G)** and IFN-g **(H)** in male and female, respectively, LOAD1.*Plcg2^M28L^* mice. The TNF-a was elevated in the HFD LOAD1.*Plcg2^M28L^* males only **(I)**. Statistical analysis was completed using an ANOVA followed by Tukey’s *post hoc* tests. *P* < 0.05 is considered significant.

Based on the specific interaction between the LOAD1.*Plcg2^M28L^* mice and the HFD in microglia density, we sought to identify cytokines in the brain or periphery that may be driving this interaction. Analysis of cytokines in the brain revealed increases of IL-1β in HFD fed-LOAD1 animals (Figure 4A); however, the same changes were not observed in the LOAD1.*Mthfr*^677C >^ *^T^* animals. In addition, a significant increase in TNF-a was observed in LOAD1 males but not in females (Figure 4C). No changes in IFN-γ were observed in the LOAD1. *Mthfr*^677C >^ *^T^* study.

In LOAD1.*Plcg2^M28L^*, we found significant reductions in several proinflammatory cytokines (IL-1β, IFN-γ) in the brain regardless of diet which was inversely correlated with plasma (Figures 4D–F). Female LOAD1 animals had an increase in plasma IL-1β in response to an HFD that was not observed in males. In contrast, in the plasma of LOAD1.*Plcg2^M28L^* mice, we found a significant increase in TNF-a (Figure 4I) in mice fed on HFD. However, there was a reduction in IL-1β (LOAD1.Plcg2*^M28L^* males) and IFN-γ (LOAD1.Plcg2*^M28L^* females), suggesting that there is a difference in the peripheral and central immune responses to an HFD.

### Interaction Between Risk Variant *Plcg2^M28L^* and HDF Correlates With AMP-AD Modules Enriched With Inflammatory- and Neuronal-System Associated Pathways

**FIGURE 5:**
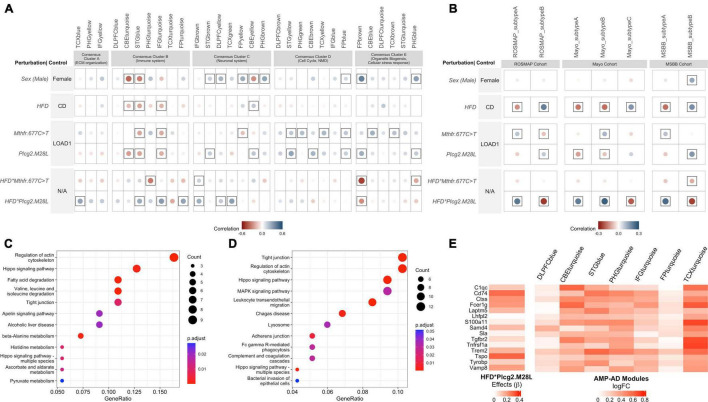
Interaction between HFD and *Plcg2^M28L^* in mice exhibits transcriptional changes in immune function similar to human LOAD. **(A)** Correlation between the effect of each mouse perturbation and 30 human co-expression modules. Each column represents one of the 30 human co-expression modules identified in seven different brain regions: the dorsolateral prefrontal cortex (DLPFC), superior temporal gyrus (STG), frontal pole (FP), parahippocampal gyrus (PHG), temporal cortex (TCX), inferior frontal gyrus (IFG), and cerebellum (CBE). These modules are grouped into five consensus clusters with similar gene content across the multiple studies and brain regions. Note that HFD*Plcg2.M28L and HFD*Mthfr.C677T results denote interaction effects separated from the individual effects of diet and variants. Controls for corresponding rows were therefore labeled N/A as the control is not strictly defined. Circles within a square correspond to significant (*p* < 0.05) positive (blue) and negative (red) Pearson’s correlation coefficients. The color intensity and size of the circles are proportional to Pearson’s correlation coefficient. **(B)** Correlation between the effect of each mouse perturbation and molecular subtypes of LOAD. The columns represent the two molecular subtypes associated with LOAD in the Religious Orders Study and the Memory and Aging Project (ROSMAP) cohort, three molecular subtypes associated with LOAD in the Mayo cohort, and two molecular subtypes associated with LOAD in the Mount Sinai Brain Bank (MSBB) cohort (Milind et al., 2020). The effects of interaction between HFD and *Plcg2^M28L^* in mice significantly correlate with the inflammatory subtypes of LOAD (Subtypes A) in each of the cohorts. Circles within a square correspond to significant (*p* < 0.05) positive (blue) and negative (red) Pearson’s correlation coefficients. **(C)** Kyoto Encyclopedia of Genes and Genomes (KEGG) Pathway enrichment analysis (FDR adjusted *p* < 0.05) of genes exhibiting directional coherence between the effects of interaction between HFD and *Plcg2^M28L^* in mice and ECM organization related AMP-AD modules in Consensus Cluster A. **(D)** KEGG Pathway enrichment analysis (FDR adjusted *p* < 0.05) of genes exhibiting directional coherence between the effects of interaction between HFD and *Plcg2^M28L^* in mice and immune-related AMP-AD modules in Consensus Cluster B. **(E)** Identification of genes exhibiting directional coherence for the interaction between HFD and *Plcg2^M28L^* in mice and change in the expression of immune-associated AMP-AD modules in Consensus Cluster B, including microglia-related genes listed.

Hallmark pathologies, such as amyloid and tau accumulation and neurodegeneration have been used widely to align mouse models to human AD. However, more recently, molecular approaches have been developed by MODEL-AD (Preuss et al., 2020) and others that allow for a more precise assessment of the relevance of mouse models to the molecular changes observed in human LOAD (Allen et al., 2016; Mostafavi et al., 2018; Wang et al., 2018; Wan et al., 2020). Here, we correlated the effect of each mouse perturbation (sex, HFD, genetic variants, and interaction between variants and HFD) with 30 human AMP-AD co-expression modules (Wan et al., 2020). These 30 modules, derived from different brain regions and study cohorts, were grouped into five “consensus clusters” based on similar gene content and repeated signals in the multiple regions. Both risk variants, *Mthfr*^677C >^ *^T^* and *Plcg2^M28L^* exhibited significant positive correlations (*p* < 0.05) with cell cycle and myelination-associated modules in Consensus Cluster D and cellular stress-response associated modules in Consensus Cluster E (Figure 4A). Moreover, *Plcg2^M28L^* displayed significant positive correlation (*p* < 0.05) with neuronal system-associated modules in Consensus Cluster C (Figure 5A). Furthermore, *Plcg2^M28L^* displayed significant negative correlation (*p* < 0.05) with immune-related modules in Consensus Cluster B, while interaction between *Plcg2^M28L^* and HFD displayed significant positive correlation (*p* < 0.05), suggesting interaction with HFD increased neuroinflammation in mice carrying the *Plcg2^M28L^* risk variant (Figure 5A). In addition, an interaction between *Plcg2^M28L^* and HFD also exhibited significant positive correlation (*p* < 0.05) with extracellular matrix organization- related module in Consensus Cluster A and neuronal system-associated modules in Consensus Cluster C (Figure 5A). Overall, we observed AD relevant phenotypes in mice for an interaction between HFD and *Plcg2^M28L^* risk variant.

We also correlated the effect of perturbation of each mouse (HFD, genetic variants, and interaction between variants and HFD) with 30 human AMP-AD co-expression modules (Wan et al., 2020) for both sexes, separately. For both male and female mice, we observed a significant positive correlation between some of the immune-related modules in Consensus Cluster B and interaction between *Plcg2^M28L^* and HFD (Supplementary Figures 2A,B). However, the effects were stronger in females as we observed a significant positive correlation (*p* < 0.05) with immune modules associated with the PHG brain region as well in females, which was not significant (*p* > 0.05) in male mice.

Genes exhibiting directional coherence between the effects of interaction between HFD and *Plcg2^M28L^* in mice and change in expression in AMP-AD modules in Consensus Clusters A and B, respectively, were extracted. A total of 99 genes were extracted that exhibited directional coherence for interaction between HFD and *Plcg2^M28L^* in mice and change in expression in AMP-AD modules in Consensus Cluster A, such as *Aldh2, Aldh6a1, Aldh7a1, lamb2, Rab31*, and *Sox9* (Supplementary File 3). We identified a total of 192 genes that exhibit a directional coherence between interaction with HFD and *Plcg2^M28L^* in mice and a change in the expression in immune-associated AMP-AD modules in Consensus Cluster B, including microglia-related genes, such as Trem2, Tyrobp, Cldn5, and C1qc (Figure 5E and Supplementary File S3). To elucidate the role of these disease-related genes, we performed a KEGG pathway enrichment analysis. Genes that showed directional coherence for the interaction between HFD and *Plcg2^M28L^* in mice and human co-expression modules in Consensus Cluster A were enriched for “Regulation of actin cytoskeleton,” “fatty acid degradation,” and multiple “metabolism” associated pathways (Figure 5C). Genes that showed directional coherence for interaction between HFD and *Plcg2^M28L^* in mice and human co-expression modules in Consensus Cluster B were enriched for multiple KEGG pathways, such as “Tight junction,” “Hippo signaling pathway,” “Lysosome,” and “phagocytosis” pathways (Figure 5D).

### Interaction Between Risk Variant *Plcg2^M28L^* and HFD Significantly Correlates With Inflammatory LOAD Subtypes

Next, to identify variants that resemble the inflammatory and non-inflammatory subtypes in human patients, we correlated the effect of each variant (sex, HFD, genetic variants, and interaction between variants and HFD) with inflammatory and non-inflammatory subtypes associated with LOAD in the ROSMAP, MSBB, and Mayo cohorts (Allen et al., 2016; De Jager et al., 2018, Wang et al., 2018). The effect of the *Mthfr*^677C >^ *^T^* variant showed a significant positive correlation (*p* < 0.05) with the inflammatory subtypes across all three cohorts, while the effect of *Plcg2^M28L^* and HFD showed a significant positive correlation (*p* < 0.05) with the non-inflammatory subtype B in the ROSMAP and MSBB cohorts (Figure 4B). Notably, the interaction between HFD and *Plcg2^M28L^* risk variant showed a strong significant positive correlation (*p* < 0.05) with the inflammatory subtypes across all three cohorts, while no significant correlation was observed for interaction between HFD and *Mthfr*^677C >^ *^T^* risk variant (Figure 5B).

### GESA Identified Upregulation of Multiple Pathways of LOAD1.Plcg2*^M28L^* HFD Animals but Not Mthfr^677C >^ *^T^*

Further, Nanostring AD panel genes were ranked based on regression coefficients calculated for each factor, and GSEA (Subramanian et al., 2005) was performed (refer to section “Materials and Methods”). The GSEA identified upregulation of Alzheimer’s disease, focal adhesion pathways in presence of each perturbation except *Mthfr*^677C >^ *^T^* (Supplementary Figure 2). Multiple immune-related pathways were upregulated and synaptic associated pathways, such as GABAergic synapse, and axon guidance were downregulated in the presence of HFD and interaction between HFD and *Plcg2^M28L^* (Supplementary Figure 1). Moreover, PCA using gene set enrichment score (NES) revealed HFD as the main effect in mice. We observed clear discrimination between genetic variants with and without interaction with HFD along the first principal component (accounting for around 59% of total variation) (Supplementary Figure 2D). The second principal component accounts for 25% of the total variance and separated *Mthfr*^677C >^ *^T^* and interaction between HFD and *Plcg2^M28L^* factors from other factors (Supplementary Figure 2).

### Increased Brain Glycolysis and Perfusion in *Plcg2^M28L^* HFD Animals

**FIGURE 6:**
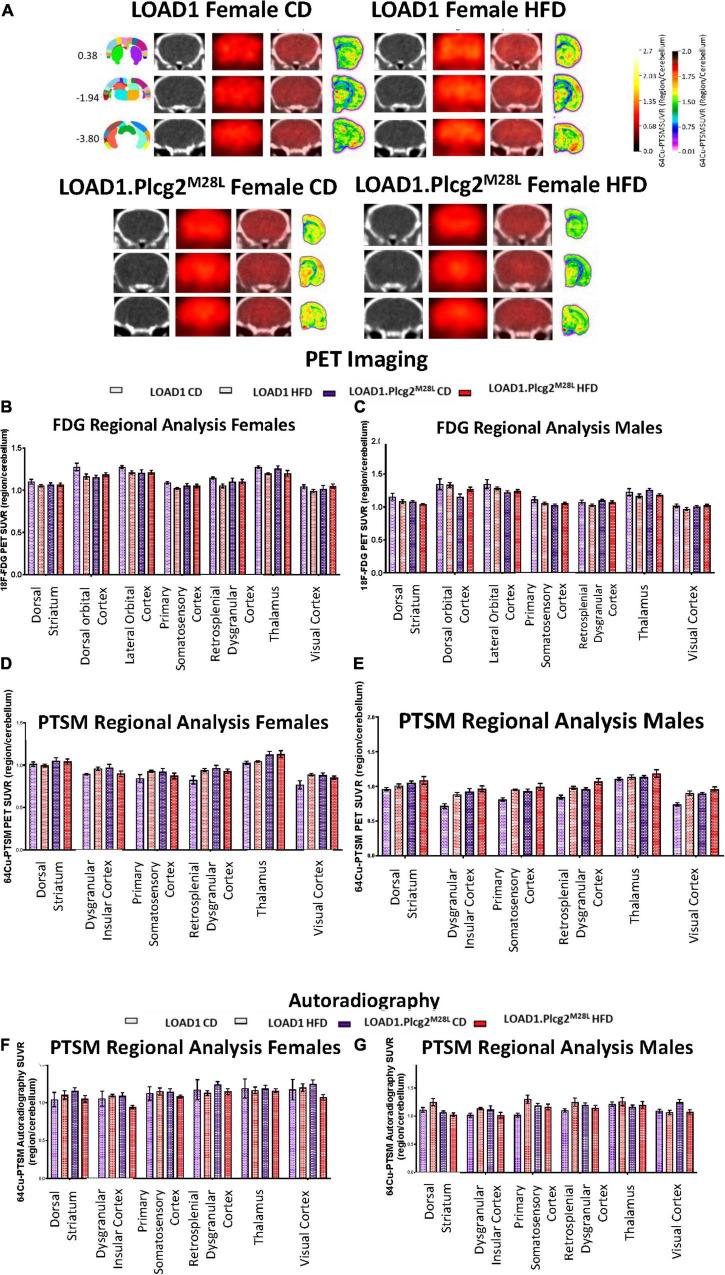
*In vivo* PET/CT imaging of LOAD1.Plcg2*^M28L^* fed with HFD. Representative images for ^64^Cu-PTSM PET/CT and autoradiography of randomly selected 12-months old female LOAD1.Plcg2*^M28L^* mice following 10-month HFD treatment **(A)**. In all cases, images are presented as standardized uptake value ratios (SUVRs) to the cerebellum. Representative bregma image panel presented as average CT (left), PET (center-left), Fused (center-right), and Autoradiography (right). Data presented are the brain regions that explain 80% of the variance determined using Principal Component Analysis (PCA) in brain glycolysis **(B,C)** and brain perfusion **(D,E)** in both females **(B,D)** and males **(C,E)**. Following the terminal ^64^Cu-PTSM scans, the brains were subjected to autoradiographic analysis **(F–G)**. Data presented are the brain regions that explain 80% of the variance.

Due to the interaction between HFD and *Plcg2^M28L^* leading to a transcriptomic inflammatory signature as well as an alteration in tight junctions, it was important to determine if there were any corresponding functional deficits. To visualize alterations in regional glycolysis and perfusion, we performed *in vivo* PET/CT imaging using ^18^F-FDG (Figures 6A–C) and ^64^Cu-PTSM (Figures 6D,E), respectively. Findings were confirmed using autoradiography. PCA comparing sex, genotype, and age determined 15 of the 27 brain regions that explained 80 percent of the variance in brain glycolysis in all mice and all conditions (Figures 6B,C). Interestingly, an HFD mice show overall increased glucose uptake compared to CD mice. Utilizing PCA analysis again for blood flow analysis, 13 of the 27 brain regions explained 80 percent of the variance in blood flow in all mice and all conditions (Figures 6D,E). Both tracers were utilized in the same animals; therefore, autoradiographic studies were only performed at the terminal scan (^64^Cu-PTSM; Figures 6F,G). Interestingly, an HFD mice showed overall increased perfusion compared to CD mice. Several regions that showed increased glucose metabolism and increased blood perfusion in a genotype-dependent manner are involved in memory and behavior. These data support a functional consequence of the observed interaction between HFD and *Plcg2^M28L^* by IHC and transcriptomics.

## Discussion

Late-onset AD is a complex disorder that is caused by a combination of genetic and environmental risk factors. However, the specific combinations of genetic and environmental factors that increase the risk for LOAD are not well understood. In fact, it is likely that these vary between individuals, making predictions of risk for LOAD based on their genetics and exposures imprecise. Previous studies support this, with E4FAD (*APOE4*+5xFAD) mice showing increased amyloid deposition, specifically more compact plaques, compared to E3FAD (*APOE3*+5xFAD) mice (Youmans et al., 2012). To further explore gene by diet effects, in our study, three new mouse models carrying different genetic risk factors for LOAD were exposed to HFD for10 months (from 2 to 12 months) to test the hypothesis that the reported effects of an HFD will vary depending on the genetic context. The results supported our hypothesis, with mice homozygous for the *Plcg2^M28L^* risk variant on a LOAD1 background showing more extreme AD phenotypes compared to either LOAD1 mice or LOAD1 mice homozygous for *Mthfr*^677C >^ *^T^*. To our knowledge, this is the first study to show that specific LOAD risk variants interact with HFD to exacerbate LOAD phenotypes whereas others do not.

Diet was provided from 2 to 12 months to model long-term exposure, from young adult to midlife, in the human population. However, our study was not designed to determine whether the changes are reversible. It is common that during an individual’s lifetime, diet is varied, particularly as a part of a weight loss program. People may go through periods of healthy eating or short-term extreme “fad” diets, such as the South Beach diet (Agatston, 2003) or the anti-inflammatory Whole 30 diet (Hartwig, 2015). However, although the benefits of changes in diet are observed for overt measures of health, such as weight, cholesterol, and blood pressure, the effects of changes in diet on the brain are not commonly tracked. Given the similarity between the effects of an HFD in humans and mice, studies where diet is varied during a lifetime, considering factors, such as time on the diet and age, from which HFD exposure began, should be performed. Here, the widely used 45% HFD (ResearchDiet feed D12451i) was used to mimic DIO. This allowed for the effects of DIO to be determined within the 12 months of this study and further studies would be needed to assess long-term effects in old age. In addition, B6 mice do not develop overt type 2 diabetes when fed with an HFD, but they are a model for early stages of the disease with phenotypes that include obesity, mildly elevated non-fasting blood glucose, increased serum glucose, glucose intolerance with advancing age and elevated triglycerides, glucose, HDL, LDL, insulin, and leptin (Collins et al., 2004)^6^. Some of those changes were observed in this study (Figure 1). However, a detailed assessment of Type 2 diabetic phenotypes was not performed here. Therefore, although we suggest not likely, we cannot rule out the interaction between HFD and *Plcg2^M28L^* observed in the brain is due to increased susceptibility to Type 2 diabetes in LOAD1.*Plcg2^M28L^* compared to the other strains tested. Furthermore, the HFD used here is somewhat extreme, not many individuals consume 45% fat daily. Therefore, to further align mouse studies to the human situation, alternative diets should be tested, such as a recently developed western diet that incorporates 16% of fat that was designed to model the more common diet consumed in the western world (Graham et al., 2016; Yang et al., 2019).

In the present study, we found alterations in several peripheral cytokines including IL-1β, IFN-γ, and TNF-α in only the LOAD1.*Plcg2^M28L^* animals. In the LOAD1 and LOAD1. *Mthfr*^677C >^ *^T^* animals, TNF-a and IL-1b were upregulated in LOAD1 animals fed HFD, but not the LOAD1.*Plcg2^M28L^* animals. However, TNF-α, IL-1β, and IFN-γ were downregulated in LOAD1.*Plcg2^M28L^* female brains, regardless of the diet. Taken together, these data suggest the mechanisms driving the interaction between HFD and *Plcg2^M28L^* may be due to a combination of peripheral and central factors (likely involving microglia responses) that conspire to increase the alignment of brain transcriptomes to human LOAD.

The interaction between *Plcg2^M28L^* and HFD was determined using the Nanostring Mouse AD panel. These analyses indicated Plcg2*^M28L^*:HFD (interaction) resulted in changes in the expression of genes enriched for inflammatory pathways. The direction of the changes correlated with gene expression changes observed in human AD based on the AMP-AD modules (Preuss et al., 2020). Many of these “inflammation” genes are expressed by microglia including *Csf1*, *Tgfbr2*, *Fcer1g*, *Trem2*, and *Tyrobp*. PLCG2 is a critical signaling element for various immune receptors and is a key regulatory hub gene for immune signaling. The PLCG2 is expected to be important in AD due to the previous findings that suggest that a hypermorphic variant in PLCG2, rs72824905, is protective against AD risk. However, the role of PLCG2 has not yet been comprehensively explored (Tsai et al., 2021; Maguire et al., 2021). A previous study has reported that reduced PLCG2 gene expression alters microglial phenotypes in 5XFAD mice, affects plaque pathology, and drives distinct transcriptional phenotypes of microglia in the presence of amyloid pathology (Tsai et al., 2021). The HFD used here is reported to create a more inflammatory environment in the brain, causing microglia activation. Previous HFD studies have been associated with increased microglial activity in wild-type mice (Rivera et al., 2013; Wanrooy et al., 2018). In the present study, transcriptomic changes were observed in the neuroinflammation module, indicative of alterations in the microglial function. Consistent with the transcriptomic changes, this study revealed an increase in microgliosis in the hippocampus of LOAD1.*Plcg2^M28L^* mice, but not in LOAD1.*Mthfr*^677C >^ *^T^*. As with all studies involving mouse models, these findings can be further supported using alternative approaches in humans and/or cell-based models (e.g., human iPSC-derived microglia), given some reports that indicate differences in microglia responses between humans and mice (Streit et al., 2009; Navarro et al., 2018; Streit et al., 2018; Olah et al., 2020).

Recent *in vivo* imaging studies have suggested that microglia displayed higher glucose uptake than neurons and astrocytes (Xiang et al., 2021). In patients with AD, glucose uptake was measured using FDG-PET, and increases in glucose uptake were observed with an increase in the microglial activity (utilizing TSPO-PET) (Xiang et al., 2021). In the present study, we observed alterations in FDG activity in multiple brain areas in LOAD1.*Plcg2^M28L^* mice. It is possible that the predicted loss of function of this variant alters the microglial state, leading to a dampened FDG signal and reduced function, which would agree with the reduced cytokine release that was observed in the brain tissue. Although we observed increases in the microglial number in female LOAD1.*Plcg2^M28L^* mice, this was only in the hippocampus of HFD animals, suggesting a potential compensatory effect. The present study suggests that FDG-PET may provide evidence for the microglial state *in vivo*.

Interestingly, the interaction between *Mthfr*^677C >^ *^T^* and HFD did not result in gene expression changes relating to inflammation and therefore PET/CT was not performed in LOAD1.*Mthfr*^677C >^ *^T^* mice. MTHFR is a key enzyme in the folate/methionine/homocysteine pathway. Variations in MTHFR, particularly the *Mthfr*^677C >^ *^T^* variant, are associated with cardiovascular diseases, AD, and vascular dementia (Liu et al., 2010; Liew and Gupta, 2015; Rai, 2017). In humans, *MTHFR*^677C >^ *^T^* reduces liver enzyme activity resulting in a decrease in enzyme function. Common effects of this include increases in homocysteine which is reported to increase the risk for vascular inflammation and dysregulation. The MTHFR is also expressed in multiple cells in the brain, particularly in vascular-related cells, such as endothelial and vascular smooth muscle cells. Supporting a role for MTHFR in the cerebrovascular function, B6 mice homozygous or heterozygous for *Mthfr*^677C >^ *^T^* show reduced enzyme activity in both the liver and brain, elevated levels of homocysteine, cerebral blood flow deficits, reduced collagen 4 in the brain, and neurovascular damage by electron microscopy (Reagan et al., 2021). However, unlike *Plcg2^M28L^*, a function for MTHFR in microglia has not been reported, further suggesting the interaction between *Plcg2^M28L^* and HFD is driven by changes in microglial function.

Despite the increased alignment at the gene expression of LOAD1.*Plcg2^M28L^* on an HFD to human LOAD compared to strain-matched mice fed on CD, LOAD1.*Plcg2^M28L^* mice fed on HFD still lack amyloid-beta plaques. This is not surprising as mice require mutations or engineering of the human sequence to drive plaque deposition, neither of which are present in these mice. Notably, this allows us to study the effect of LOAD genetic risk x aging in the absence of amyloid pathology. Also, neuronal loss was not detected in LOAD1.*Plcg2^M28L^* mice on an HFD, at least in the brain regions assessed up to 12 months of age, suggesting additional pathways/processes need to be perturbed likely in combination with extended aging to further align these models to human LOAD. The presence of age-dependent amyloid accumulation would be expected to further modify LOAD phenotypes and possibly result in neuronal cell loss. Therefore, a humanized amyloid-beta (hAb) allele has been created by MODEL-AD and added to LOAD1 (to create LOAD2; B6.*APOE4*.*Trem2^R47H^*.hAb) and to LOAD1.*Plcg2^M28L^* and LOAD1.*Mthfr*^677C >^ *^T^* to create LOAD2.*Plcg2^M28L^* and LOAD2.*Mthfr^C677T^*, respectively. Male and female test and control mice are provided with HFD and aged from 18 to 24 months to determine the long-term effects of combining *APOE4*, *Trem2^R47H^*, *Plcg2^M28L^*, or *Mthfr*^677C >^ *^T^* risk variants in combination with aging and the more amyloidogenic humanized hAb.

Despite the fact that all mice carried the *APOE4* and *Trem2^R47H^* variants, linear modeling identified the specific effects of the *Plcg2^M28L^* and *Mthfr*^677C >^ *^T^* variants and the *Plcg2^M28L^*:HFD and *Mthfr*^677C >^ *^T^*:HFD interactions. However, data now suggest that although APOE4 and *TREM2^R47H^* are strong genetic risk factors for LOAD in humans, *TREM2^R47H^* may be reducing the effect of the APOE4 variant when present together (Kotredes et al., 2021). This may be due in part to the reduced expression levels of *Trem2* in *Trem2^R47H^* mice caused by a cryptic splice site that results in an aberrant splice form (JAX#27918). MODEL-AD has created a new *Trem2^R47H^* allele (Trem2*R47H ^HSS^) that incorporates a human splice site (HSS) and restores *Trem2* expression to normal levels, at least in young wild type B6 mice (JAX#33781). Future studies will incorporate APOE4 or Trem2*R47H^HSS^ in combination with the hAb allele and a recently created humanized, MAPT-GR (JAX # 35398 and 33668) to study the interaction between HFD and Tau pathology in the context of LOAD. These mice express the human MAPT (H1 or H2 haplotype, respectively) and MAPT-AS1 transcripts and the typical MAPT protein isoforms.

In summary, combining genetic and environmental risk factors (e.g., HFD) leads to better translational and preclinical models of LOAD. The interactions we have observed here that correlate with LOAD in the human population suggest that the effects of an HFD are genotype-specific and further investigation is needed to resolve the mechanistic interactions between genetics and diet.

## Data Availability Statement

The datasets presented in this study can be found in online repositories. The names of the repository/repositories and accession number(s) can be found in the article/Supplementary Material.

## Ethics Statement

The animal study was reviewed and approved by Indiana University IACUC; The Jackson Laboratory AUC.

## Author Contributions

AO, KK, and GH contributed to the design of the study and wrote the manuscript. RP, GC, and MS contributed to data analysis and interpretation of data. AR, CI, BP, CL, DB, PL, DS, AT, SP, AB, KE, RS, JM, JP, and LF ran assays and completed data analysis for these studies. GH, PT, SR, and BL oversaw the study. All authors contributed to the article and approved the submitted version.

## Conflict of Interest

The authors declare that the research was conducted in the absence of any commercial or financial relationships that could be construed as a potential conflict of interest.

## Publisher’s Note

All claims expressed in this article are solely those of the authors and do not necessarily represent those of their affiliated organizations, or those of the publisher, the editors and the reviewers. Any product that may be evaluated in this article, or claim that may be made by its manufacturer, is not guaranteed or endorsed by the publisher.

## Footnotes

1
https://adknowledgeportal.synapse.org/Explore/Studies/DetailsPage?Study=syn21595255

2
https://www.labsupplytx.com/wp-content/uploads/2012/10/5K52.pdf

3
https://researchdiets.com/formulas/d12451

4
https://www.synapse.org/#!Synapse:syn14237651

5
https://reactome.org/

6
https://www.jax.org/jax-mice-and-services/strain-data-sheet-pages/phenotypeinformation-380050
