# Supplementary material for: Plcg2M28L Interacts With High Fat/High Sugar Diet to Accelerate Alzheimer’s Disease-Relevant Phenotypes in Mice
Source: Front Aging Neurosci. 2022 Jun 24;14:886575. doi: 10.3389/fnagi.2022.886575 (PMC9263289; doi:10.3389/fnagi.2022.886575)
Supplement: Supplementary file 1 [file Data_Sheet_1.PDF]

A

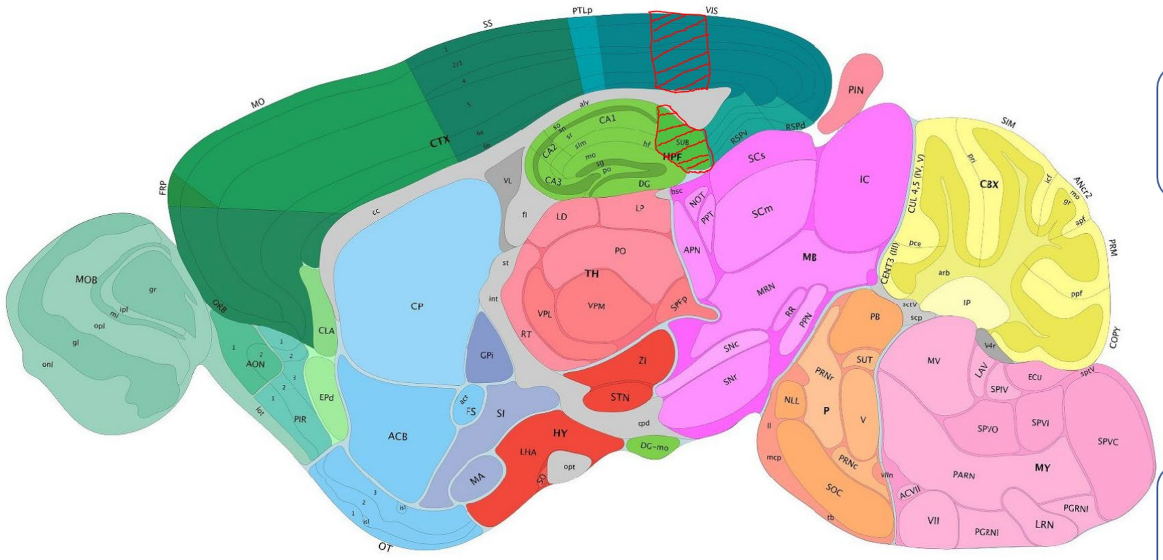

B

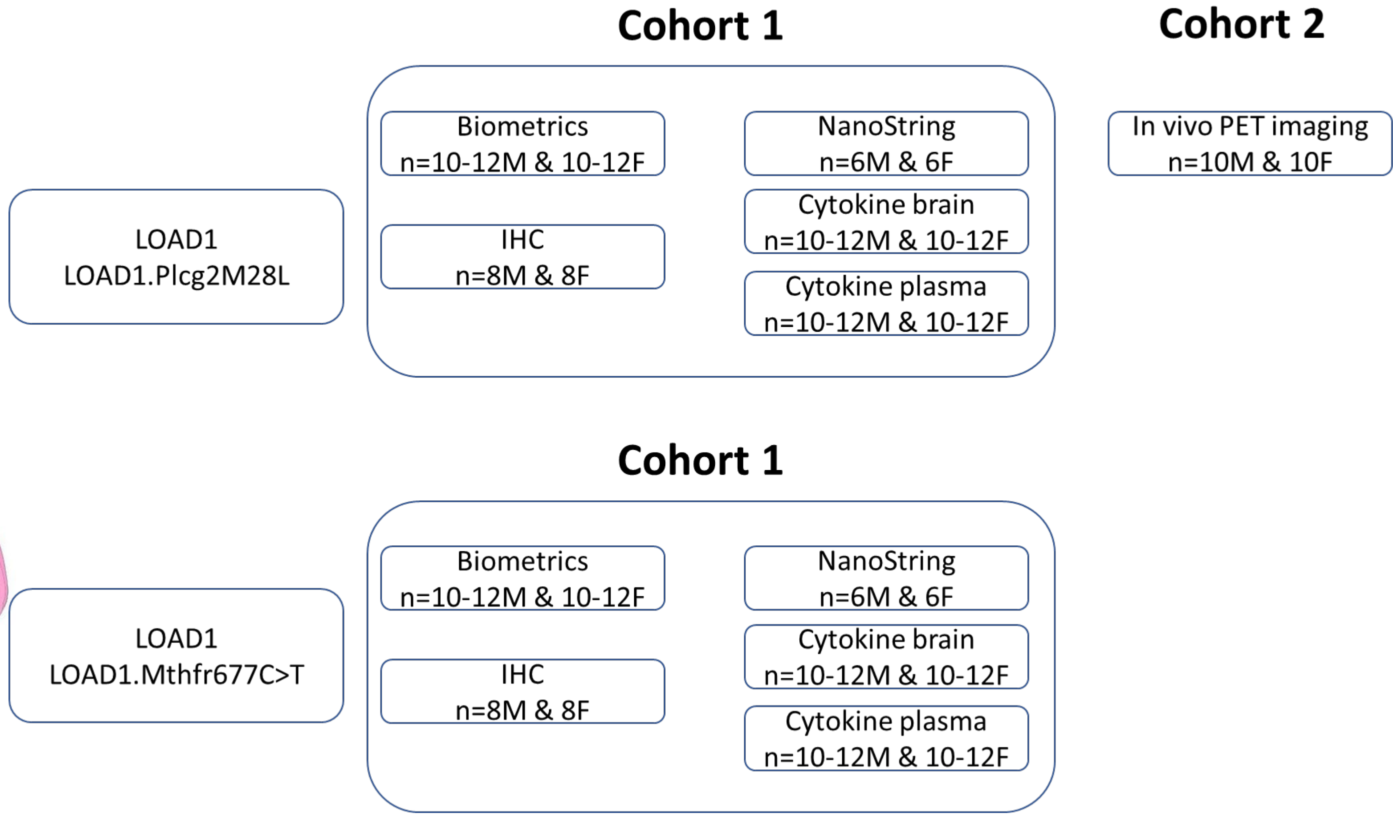
Cohort 1
LOAD1
LOAD1.Plcg2M28L
Biometrics
n=10-12M & 10-12F
IHC
n=8M & 8F
NanoString
n=6M & 6F
Cytokine brain
n=10-12M & 10-12F
Cytokine plasma
n=10-12M & 10-12F
Cohort 2
In vivo PET imaging
n=10M & 10F
Cohort 1
LOAD1
LOAD1.Mthfr677C>T
Biometrics
n=10-12M & 10-12F
IHC
n=8M & 8F
NanoString
n=6M & 6F
Cytokine brain
n=10-12M & 10-12F
Cytokine plasma
n=10-12M & 10-12F
