# Supplementary figures and images for: Plcg2M28L Interacts With High Fat/High Sugar Diet to Accelerate Alzheimer’s Disease-Relevant Phenotypes in Mice
Source: Front Aging Neurosci. 2022 Jun 24;14:886575. doi: 10.3389/fnagi.2022.886575 (PMC9263289; doi:10.3389/fnagi.2022.886575)

**Figure S1.**

**A.**

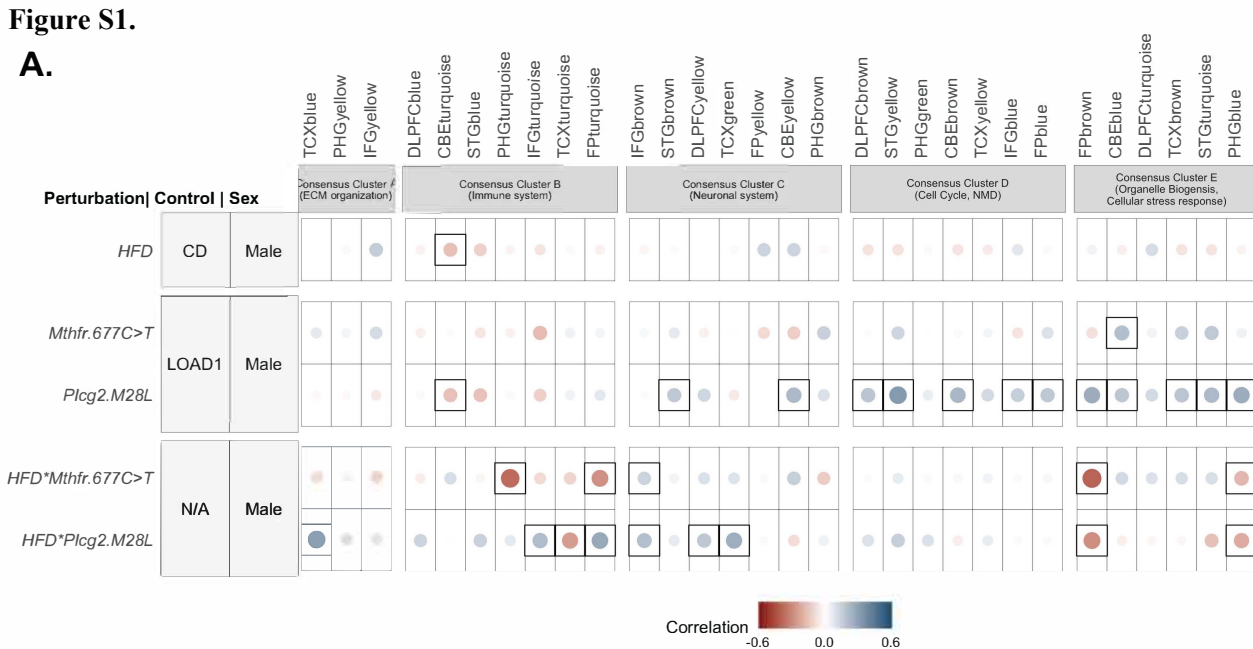

**B.**

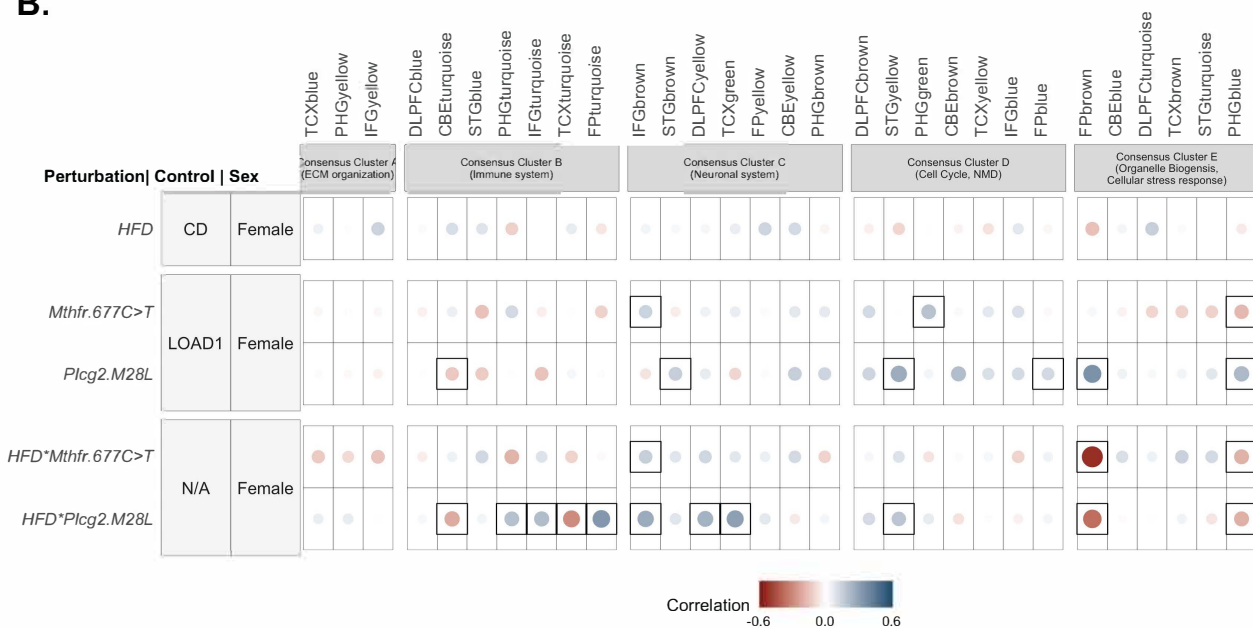

**C.**

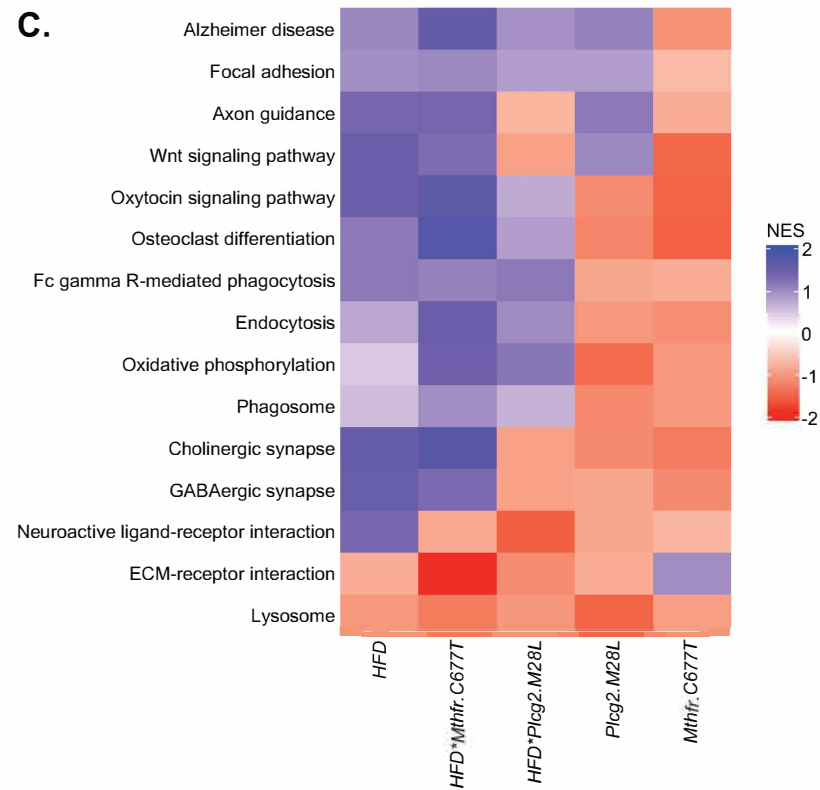

**D.**

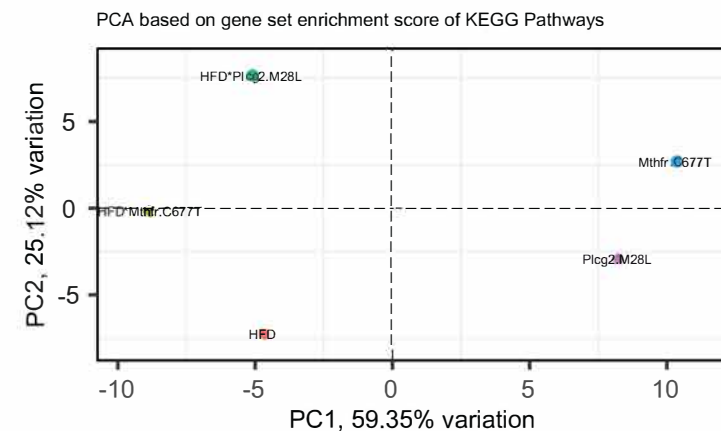

Supplement: Supplementary file 2 [file Data_Sheet_2.PDF]
